# Improving Prognosis of Surrogate Assay for Breast Cancer Patients by Absolute Quantitation of Ki67 Protein Levels Using Quantitative Dot Blot (QDB) Method

**DOI:** 10.3389/fonc.2021.737781

**Published:** 2021-09-17

**Authors:** Junmei Hao, Yan Lyu, Jiarui Zou, Yunyun Zhang, Shuishan Xie, Lili Jing, Fangrong Tang, Jiahong Lyu, Wenfeng Zhang, Jianbo Zhang, Xunting Wang, Kuisheng Chen, Jiandi Zhang

**Affiliations:** ^1^Department of Pathology, Yantai Affiliated Hospital of Binzhou Medical University, Yantai, China; ^2^Yantai Quanticision Diagnostics, Inc., Yantai, China; ^3^Department of Imaging, Linglong Yingcheng Hospital, Zhaoyuan, China; ^4^Department of Pathology, The First Affiliated Hospital of Zhengzhou University, Henan Province Key Laboratory of Tumor Pathology, Zhengzhou, China

**Keywords:** surrogate assay, adjusted surrogate assay, Ki67, QDB, quantitative, FFPE

## Abstract

**Background:**

Immunohistochemistry (IHC)-based surrogate assay is the prevailing method in daily clinical practice to determine the necessity of chemotherapy for Luminal-like breast cancer patients worldwide. It relies on Ki67 scores to separate Luminal A-like from Luminal B-like breast cancer subtypes. Yet, IHC-based Ki67 assessment is known to be plagued with subjectivity and inconsistency to undermine the performance of the surrogate assay. A novel method needs to be explored to improve the clinical utility of Ki67 in daily clinical practice.

**Materials and Methods:**

The Ki67 protein levels in a cohort of 253 specimens were assessed with IHC and quantitative dot blot (QDB) methods, respectively, and used to assign these specimens into Luminal A-like and Luminal B-like subtypes accordingly. Their performances were compared with the Kaplan–Meier, univariate, and multivariate survival analyses of the overall survival (OS) of Luminal-like patients.

**Results:**

The surrogate assay based on absolutely quantitated Ki67 levels (cutoff at 2.31 nmol/g) subtyped the Luminal-like patients more effectively than that based on Ki67 scores (cutoff at 14%) (Log rank test, *p* = 0.00052 *vs*. *p* = 0.031). It is also correlated better with OS in multivariate survival analysis [hazard ratio (HR) at 6.89 (95% CI: 2.66–17.84, *p* = 0.0001) *vs*. 2.14 (95% CI: 0.89–5.11, *p* = 0.087)].

**Conclusions:**

Our study showed that the performance of the surrogate assay may be improved significantly by measuring Ki67 levels absolutely, quantitatively, and objectively using the QDB method.

## Introduction

Microarray analysis of global gene profiling (GEP) of breast cancer tissues leads to the identification of the four intrinsic subtypes: luminal, Her2-like, basal-like, and normal-like subtypes (1, 2). The Luminal-like patients are further separated into Luminal A (LumA) and Luminal B (LumB) patients. Its distinction is based on the proliferation status among Luminal-like patients (3). LumA patients are associated with better clinical outcomes than that of LumB patients.

While the benefits of endocrine therapy to Luminal-like patients have been well established, there is increased recognition that chemotherapy may not be a necessary part of adjuvant therapy for all luminal-like patients (4–7). It is well accepted now that LumA patients benefit less from chemotherapy than LumB patients (5). Thus, chemotherapy may be spared from many LumA patients, considering its strong side effects. This concept has been well accepted with several GEP-based genetic tests developed to identify Luminal subtype patients who may be spared of chemotherapy (5).

Yet, GEP-based genetic tests remain inaccessible to a lot of patients worldwide (5). As an alternative, an immuno-histochemistry (IHC)-based surrogate assay has been used extensively all over the world. Based on the 2013 St. Gallen Consensus, the patients are categorized into Luminal-like, Her2 positive (non-luminal), and Triple negative (ductal) subtypes based on IHC assessment of ER, PR, Her2, and Ki67 (5). The Luminal-like patients are further separated into LumA patients with ER+, Her2−, PR score ≥20%, and Ki67 score <14% (5). The LumB patients are composed of Her2− (LumB_1_) and Her2+ (LumB_2_) subgroups. LumB_1_ patients are ER+, Her2−, with Ki67 score ≥14%, or PR score <20%. LumB_2_ patients are ER+ and Her2+, regardless of the Ki67 and PR statuses (5). In 2015, the cutoff of Ki67 score to separate LumA from LumB was adjusted to 20% (6).

Clearly, the expression level of Ki67, the well-accepted protein biomarker of tumor progression, is the factor to consider when deciding if a Luminal-like patient may be spared of chemotherapy (5, 6). Currently, Ki67 levels are mainly assessed through IHC, a method known to be associated with subjectivity and inconsistency. Among all four biomarkers used in the surrogate assay, the standardization of Ki67 assessment may be considered most difficult. Intensive efforts have been devoted to the standardization of this biomarker; yet, there remains quite a distance away from its realization (8–12). In fact, a group of renowned oncologists in the breast cancer field [International Ki67 Work Group (IKWG)] declared that the IHC-based Ki67 “has limited value for treatment decisions due to questionable analytical validity” (13). Even the latest recommendations from IKWG has issue of applicability in daily clinical practice (14, 15). Apparently, other alternative methods need to be explored to address this challenge.

Recently, a high-throughput immunoassay, quantitative dot blot (QDB) method, was developed to measure protein levels at the tissue level objectively, quantitatively, and absolutely (16–18). In a certain sense, this method is an enzyme-linked immunosorbent assay (ELISA)-like assay for formalin-fixed paraffin-embedded (FFPE) specimens, considering ELISA is not suitable for FFPE specimens due to heavy crosslinking of tissue proteins (19).

In most cases, QDB can convert an IHC assay into a QDB assay directly utilizing the same clinically validated diagnostic antibodies for IHC. Like ELISA, various internal controls are included in the analytical process to ensure the accuracy and consistency of the assay. Admittedly, the unmatched accuracy and consistency of QDB method over those of IHC is at the loss of morphological information. Undoubtedly, these two methods need to be combined to offer a comprehensive picture of the distribution and expression of a protein biomarker in FFPE specimens.

In this study, we attempted to measure Ki67 levels absolutely, quantitatively, and objectively using the QDB method in FFPE specimens to improve the consistency and accuracy of Ki67 measurement in clinical practice. A QDB-based assay was developed using MIB1, the recommended Ki67 antibody for IHC, and a recombinant Ki67 protein (20). The specimens were separated into luminal A (LumA*_q_*) and luminal B subtypes (LumB*_q_*) based on the absolute values of Ki67 in these specimens in a retrospective study (**Table 1**). The prognosis of this adjusted surrogate assay was compared with that of the IHC-based surrogate assay to demonstrate the potential of objective measurement of Ki67 protein levels for subtyping of Luminal-like tumors in daily clinical practice.

**Table 1:** Clinicopathological characteristics of 155 Luminal-like breast cancer specimens.

Characteristics	Number of Cases (%)
**Age**	
<50	69 (44.52)
≥50	86 (55.48)
**Treatment Type**	
Endo^1^	2 (1.29)
Chemo^2^	90 (58.06)
Endo&Chemo^3^	41 (26.45)
Other^4^	22 (14.19)
**Pathological Lymph Node Status, pN**	
pN0	75 (48.39)
pN1	56 (36.13)
pN2	10 (6.45)
pN3	8 (5.16)
Unknown	6 (3.87)
**Pathological Tumor Size, pT**	
pT1	54 (34.84)
pT2	91 (58.71)
pT3	7 (4.52)
Unknown	3 (1.94)
**Histological Grade**	
II	83 (53.55)
III	52 (33.55)
Not applicable	20 (12.9)
**Surrogate Assay** (14% Ki67 cutoff)	
LumA*_i_*	66 (42.58)
LumB*_i_*	89 (57.42)
LumB_1_ *_i_*	70 (45.16)
LumB_2_ *_i_*	19 (12.26)
**Surrogate Assay** (20% Ki67 cutoff)	
LumA*_i_*	76 (49.03)
LumB*_i_*	79 (50.97)
LumB_1_ *_i_*	60 (38.71)
LumB_2_ *_i_*	19 (12.26)

The treatment plan was developed by physicians by following the guidance issued by the Chinese Anti-Cancer Association (CACA) in 2007 (21) at physician’s discretion. 1: Tamoxifen or toremifene citrate tablet; 2: CAF (cyclophosphamide, doxorubicin hydrochloride, and fluorouracil) or CMF (cyclophosphamide, methotrexate, and fluorouracil) or TAC (Doxorubicin Hydrochloride and cyclophosphamide with or followed by Docetaxel); 3: one regimen from 2 followed by one regimen from 1; 4: non-standard treatments including Chinese traditional medicine or informed refusal by patients.

## Materials and Methods

### General Reagents

All general reagents used for cell culture were purchased from Thermo Fisher Scientific Inc. (Waltham, MA, USA), including the cell culture media and culture dishes. The protease inhibitors were purchased from Sigma Aldrich (St. Louis, MO, USA). All other chemicals were purchased from Sinopharm Chemicals (Beijing, P. R. China). QDB plates were manufactured by Quanticision Diagnostics, Inc. (RTP, USA). Mouse anti-Ki67 antibody (clone MIB1) was purchased from ZSGB-BIO (Beijing, China). HRP-labeled Donkey Anti-Mouse IgG secondary antibody was purchased from Jackson Immunoresearch lab (Pike West Grove, PA, USA).

PCR reagents, restriction enzymes, and T4 DNA ligase were purchased from Takara Bio Inc. (Dalian, China). Competent cells *Escherichia coli* DH5a and BL21(DE3) were from TransGen Biotech (Beijing, China). IPTG (Isopropyl β-D-1-thiogalactopyranoside) was purchased from Solarbio (Beijing, China). Nickel-His GraviTrap affinity column was purchased from GE Healthcare.

### Purification of Recombinant Ki67 Fragment

A DNA sequence corresponding to the 1162–1254AA of human MKI67 (NCBI #: NM_002417.4) was synthesized by Sangon Biotech (Shanghai, China) and was inserted into pET-32a (+) expression vector. The plasmid was verified by sequencing and expressed in BL21 (DE3) competent cells. The cells were induced with IPTG, and total bacterial lysate was extracted in 10 ml of binding buffer (20 mM sodium phosphate, 500 mM NaCl, and 20 mM imidazole, pH 7.4) before it was loaded onto a high-affinity Ni2+ column pre-equilibrated with 10 ml of binding buffer. The recombinant protein was eluted with 3 ml of elution buffer (20 mM sodium phosphate, 500 mM NaCl, and 250 mM imidazole, pH 7.4) and dialyzed in PBS (pH 7.4) at 4°C overnight. The purity of the protein was examined by a 12% SDS-PAGE gel at 80%, and the purified protein was stored at −80°C in a small aliquot with 20% glycerol.

### Preparation of FFPE and Cell Lysates

To extract total protein, 2 × 15 μm FFPE slices were first de-paraffinized and then solubilized with lysis buffer (50 mM HEPES, 137 mM NaCl, 5 mM EDTA, 1 mM MgCl_2_, 10 mM Na_2_P_2_O_7_, 1% Triton X-100, and 10% glycerol). Total protein concentration was measured using Pierce BCA protein assay kit in accordance to the manufacturer’s instructions. BT474 and 293T cells were fixed in Formalin Solution for 30 mins before they were lysed in the same lysis buffer with protease inhibitors. The supernatants were collected after centrifugation and the total amount of proteins were measured using BCA protein assay kit by following the manufacturer’s instructions.

### Human Subjects and Human Cell Lines

The inclusion criteria for this retrospective observational study were patients diagnosed as breast cancer patients with FFPE tissue specimen available at Yantai Affiliated Hospital of Binzhou Medical University, Yantai, P. R. China from 2008 to 2013 consecutively and non-selectively. The specimen must have more than 50% tumor tissue based on H&E staining. Follow-up data were available for 221 patients (87.4%) at the last follow-up on April 1, 2019.

All the treatments that patients received in this study were adjuvant treatments. Clinical information, including age, pathological lymph node status, pathological tumor size, histological grade, type of treatments [chemotherapy (CT), endocrine therapy (ET), or chemoendocrine therapy (CET)], and results of FISH analysis, was collected from medical records. The end point was overall survival (OS) defined as the time between breast cancer surgery and death or last follow-up. All the missing values were treated as a new category. The cases lost to follow-up were not included in the analysis. Patients still alive at last study follow-up (April 1, 2019) were censored.

### IHC Analysis

IHC for ER, PR, HER2, and Ki67 was performed concurrently on serial sections with the standard streptavidin–biotin complex method with 3, 3'-diaminobenzidine as the chromogen. Staining for ER, PR, and HER2 interpretation was performed by following the Dako autostainer link 48 manual (Ft. Collins, CO). ER antibody (clone SP1) and PR antibody (clone SP2) from MXB Biotechnologies, HER2 antibody (polyclonal A0485) from Dako, and Ki67 (Clone SP6) from LBP (www.gzlbp.com) were all at 1:100 dilution after antigen retrieval in 0.05 M Tris buffer (pH 9.0) with heating to 95°C for 20 min. Biomarker expressions from IHC assays were scored by three pathologists [JMH (26), JRZ (9), and LLJ (11); the number indicated the years of professional experience] who were blinded to the clinicopathological characteristics and outcomes and who used previously established/published criteria for biomarker expression levels routinely used in daily clinical practice. Tumors were considered positive for ER if immunostaining was observed in more than 1% of tumor nuclei, as recommended by ASCO/CAP guidance. Tumors were considered positive for HER2 if either immunostaining was scored as 3+ according to HercepTest criteria or FISH test positive. Ki67 and PR were visually scored for percentage of tumor cell nuclei with positive immunostaining above the background level. The positively stained nucleus was considered a valid signal for Ki67, with the Ki67 scores determined by the average method. A panel of representative IHC images stained with Ki67 antibody was included in **Supplementary Figure S1**.

### QDB Analysis

The QDB process was described in detail elsewhere with slight modifications (16, 19). In short, the final concentration of the FFPE tissue lysates was adjusted to 0.25 μg/μl, and 2 μl/unit was used for QDB analysis as well as a serially diluted recombinant protein in triplicate. The loaded QDB plate was dried for 1 h at RT and then blocked in 4% non-fat milk for an hour. Anti-Ki67 antibody (MIB1) was diluted at 1:1,000 in blocking buffer, and incubated with QDB plate at 100 μl/well overnight at 4°C, and incubated next with a donkey anti-mouse secondary antibody for 4 h at RT. The QDB plate was inserted into a white 96-well plate pre-filled with 100 μl/well ECL working solution for 3 mins for quantification with Tecan Infiniti 200pro Microplate reader with the option “plate with cover”.

The consistency of the experiments was ensured by including two cell lysates with known Ki67 levels (BT474 and 293T) in all the experiments. The result was considered valid when the calculated Ki67 level of BT474 and 293T was within 20% of known Ki67 level at 6.3 (5.04–7.56) nmol/g and 4.4 (3.52–5.28) nmol/g, respectively. The absolute Ki67 level was determined based on the dose curve of protein standard. Ki67 level less than 25 pg (about 1.4 nmol/g) was defined as Limit of Quantitation (LOQ), and entered as 0 for data analysis. In addition, the Limit of Blank (LOB) and Limit of Detection (LOD) were defined as 1.11 and 1.24 nmol/g from multiple experiments [14]. The signal-to-noise ratio curves were also plotted in **Supplementary Figure S2**.

### Statistical Analysis

GraphPad 7 software (La Jolla, CA, USA) was used for common data analysis, including Pearson’s correlation coefficient analysis. The results were presented as mean ± SD. The survival analyses were done using R version 3.6.2 (http://www.r-project.org). The strength of the agreement among Ki67 IHC scores from three pathologists was assessed by Fleiss’s Kappa analysis.

The Ki67 levels measured by the QDB method or the IHC method were dichotomized for OS by using optimal cutoff values determined by the “surv_cutpoint” function of the “surviminer” R package, respectively, with optimized cutoff at 2.31 nmol/g for the QDB method and 14% for the IHC method accordingly. All the OS analyses were visualized by Kaplan–Meier method, and comparisons were performed by Log rank test.

Univariate Cox proportional hazard models and fitted OS were employed for hazard ratio (HR) and corresponding 95% confidence intervals (CIs) estimation. Multivariable Cox models were utilized to examine the association between subtypes and OS, adjusting for other clinical variables, such as age, pathological lymph node status, pathological tumor size, histological grade, and type of treatment. Residuals that are analogous to the Schoenfeld residuals in Cox models were used to check the proportionality assumption. *p*-values of less than.05 were considered statistically significant.

## Results

### Measurement of Ki67 Protein Levels With the QDB Method

A QDB-based high-throughput immunoassay for absolute quantitation of Ki67 levels in FFPE specimens was developed first by defining the linear range of total tissue lysates and recombinant Ki67 protein standards using MIB1. The total tissue lysates from four FFPE specimens with Ki67 score >70% were pooled together, and diluted serially to define the linear range of the assay (**Supplementary Figure S3**).

**Figure 1:**
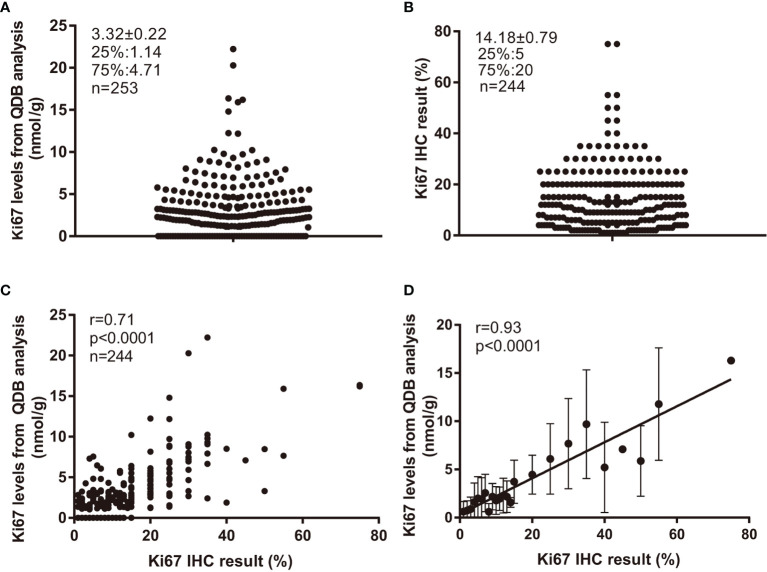
Ki67 levels in 253 FFPE specimens and their correlations with Ki67 scores from IHC analysis. Total lysate was extracted from 2 × 15 μm FFPE slices individually, and 0.5 μg/specimen was used for QDB measurement using Mouse anti-Human Ki67 monoclonal antibody (MIB1). These specimens were also assessed with IHC analysis, with each IHC-stained slide assessed by three pathologists independently. The Ki67 scores used in the study were averages of three assessments. **(A)** Distribution of quantitatively measured Ki67 levels among these specimens. **(B)** Distribution of Ki67 scores from IHC analysis among 244 specimens. **(C)** Correlation analysis of the results from QDB and IHC analyses using Pearson’s correlation analysis with *r* = 0.71, *p* < 0.0001. **(D)** These specimens were subgrouped based on their respective Ki67 scores. The subgroup averages of the Ki67 levels from QDB measurements were used for correlation analysis with Ki67 scores from IHC analysis using Pearson’s correlation analysis with *r* = 0.93, *p* < 0.0001. The results were expressed as mean ± SD.

The Ki67 levels in all 253 FFPE specimens were measured using the QDB method, and Ki67 levels were found to distribute between 0 (undetectable level) to 22.21 nmol/g, with average at 3.32 ± 0.22 nmol/g (**Figure 1A**). Based on a recent study (22), the ductal carcinoma *in situ* (DCIS), normal, and stroma tissue were not excluded from the tissue slices, as long as more than 50% of invasive tumor was presented in the slice. In this study, the potential influence of tumor infiltrating lymphocytes (TIL) was also not considered.

Among 253 specimens, 244 were provided with Ki67 scores from three pathologists assessing the same set of IHC-stained slides independently. Their Ki67 score averages were used throughout the study. We found that the highest IHC score was at 75%, and the lowest at 1%, with an average at 14.18% ± 0.79% (**Figure 1B**). Correlation analysis was performed using results from QDB and IHC methods with *r* = 0.71, *p* < 0.0001 using Pearson’s correlation analysis (**Figure 1C**). In an attempt to reduce the potential interference from the subjectivity inherently associated with IHC analysis, we also subgrouped these specimens by their IHC scores. As expected, the correlation between the subgroup averages of the absolute Ki67 levels from the QDB method with IHC scores was increased to *r* = 0.93, *p* < 0.0001 using Pearson’s correlation analysis (**Figure 1D**).

**Figure 2:**
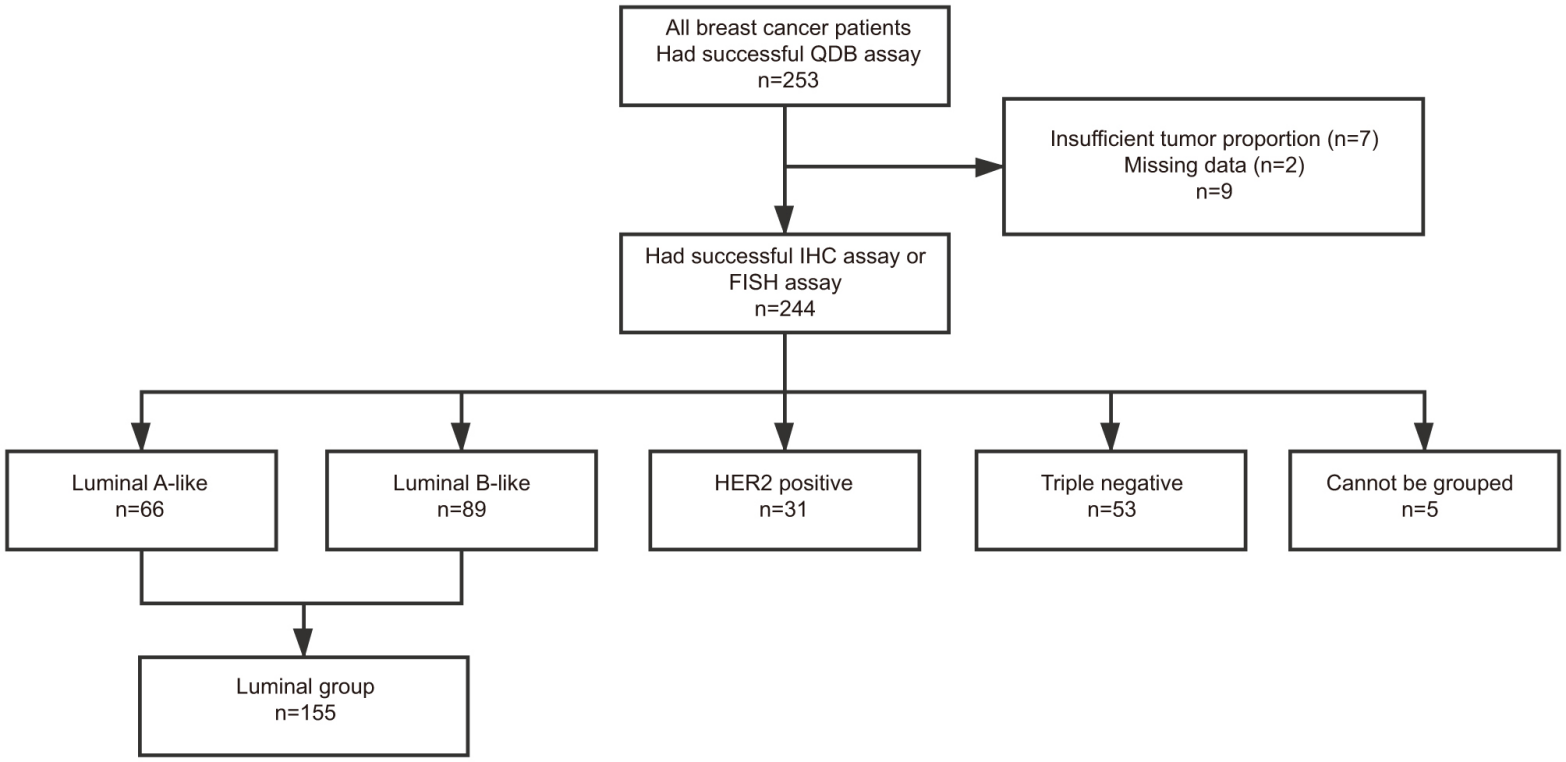
Flow diagram of patient selection for the study.

Those specimens provided with Ki67 scores were also accompanied with IHC results for ER, PR, and Her2. For specimens with a Her2 score of 2+, results from FISH analysis were used to differentiate Her2+ from Her2− specimen. Based on these information, we assigned these 244 specimens into luminal-like subtype (*n* = 155), HER2-like subtype (*n* = 31), and Triple Negative subtype (*n* = 53) based on 2013 St. Gallen consensus (5). The remaining five specimens cannot be subtyped based on this consensus (**Figure 2**).

The clinicopathological parameters of the 155 luminal-like specimens are listed in **Table 1**. All the treatments were adjuvant therapies. For all the qualified patients, the median OS time to censoring was 85 months, with the maximum at 132 months. These specimens can be further divided into 66 Luminal A-like and 89 B-like subtypes using Ki67 score at 14% as cutoff based on 2013 St. Gallen consensus (5), or 76 Luminal A-like and 79 Luminal B-like subtypes with Ki67 score at 20% as cutoff based on 2015 St. Gallen consensus (6).

### QDB-Based Adjusted Surrogate Assay *vs*. IHC-Based Surrogate Assay

To evaluate the influence of objectively quantitated Ki67 levels on the prognostic effect of the surrogate assay, we subtyped luminal A-like from luminal B-like subtypes based on absolutely quantitated Ki67 levels, using an optimized cutoff at 2.31 nmol/g. We named this method the *adjusted surrogate assay* for simplicity (**Supplementary Table S1**). The 2.31 nmol/g cutoff used in the adjusted surrogate assay was obtained using the “surv_cutpoint” function of the “suvminer” R package in combination with the OS of these patients. This proposed cutoff was validated using an independent cohort of breast cancer patients (23). In addition, we also managed to split the current cohort randomly into a training set and a validate set using RAND (“table”) function with SAS 9.4 to demonstrate its effectiveness for subtyping of Luminal-like patients (**Supplementary Figure S4**).

We also managed to obtain the optimum cutoff for Ki67 score from IHC analysis at 2.67% using the same function. However, at this value, only a small fraction of specimens were assigned to Luminal A-like subtype (*n* = 26). Therefore, Ki67 scores of 14% or 20% were evaluated as IHC cutoffs respectively based on different opinions from St. Gallen consensuses at 2013 and 2015.

**Figure 3:**
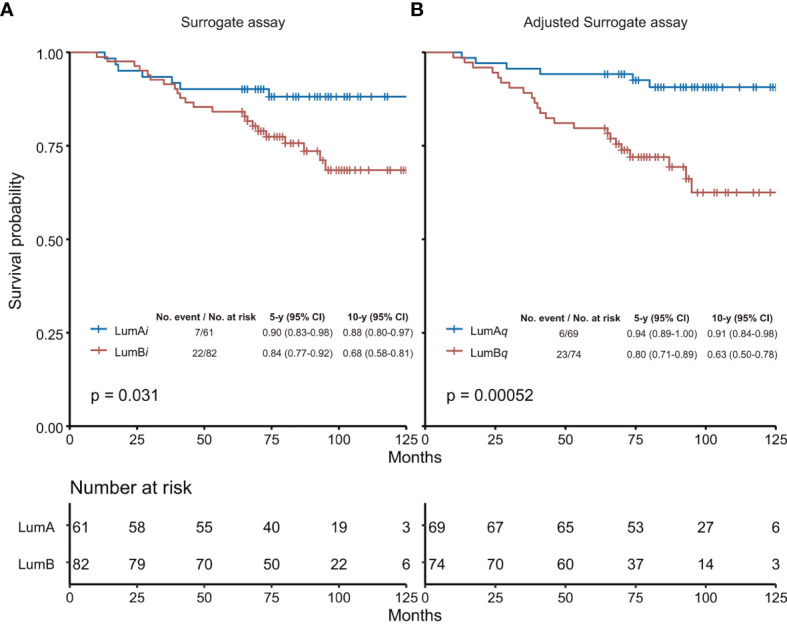
Overall survival analysis by surrogate assay **(A)** or adjusted surrogate assay **(B)**. **(A)** The Ki67 score of 14% was used as cutoff in the surrogate assay based on Recommendations from 2013 St. Gallen Consensus. **(B)** The Ki67 level of 2.31 nmol/g was used as cutoff determined by the “surv_cutpoint” function of the “surviminer” R package in the adjusted surrogate assay. The 5-year and 10-year survival probabilities, and the *p*-values from Log rank test were provided for both the surrogate assay and the adjusted surrogate assay, respectively. LumA, Luminal A-like subtype; LumB, Luminal B-like subtype; LumA*_i_* and LumB*_i_*, Luminal A-like and B-like subtypes by IHC-based surrogate assay; LumA*_q_* and LumB*_q_*, Luminal A-like and B-like subtypes by QDB-based adjusted surrogate assay; CI, confidence interval.

As shown in **Figure 3**, based on the adjusted surrogate assay, the luminal A-like subtype (LumA*_q_*) had 10-year survival probability (10y SP) at 91% *vs*. 63% for Luminal B-like subtype (LumB*_q_*), with *p* = 0.00052 from Log rank test. In contrast, 10y SP for luminal A-like subtype (LumA*_i_*) was 88% *vs*. 68% for Luminal B-like subtype (LumB*_i_*), with *p* = 0.031 from the surrogate assay with 14% as Ki67 cutoff. When Ki67 score of 20% was used as cutoff, 10y SP for LumA*_i_* was 84% *vs*. 70% for LumB*_i_*, with *p* = 0.10 (**Supplementary Figure S5**). Clearly, in this study, the surrogate assay using 14% as Ki67 cutoff performed better than that using 20%. Therefore, we chose to compare the adjusted surrogate assay with the surrogate assay using 14% as Ki67 cutoff for the rest of the studies.

The surrogate assay was compared next with the adjusted surrogate assay in univariate Cox regression analysis, and we found that the adjusted surrogate assay provided improved prognosis for Luminal-like breast cancers with HR at 4.39 (95% CI, 1.78–10.81, *p* = 0.0013) than that of the surrogate assay with HR at 2.46 (95% CI, 1.05-5.75, *p* = 0.0385) (**Supplementary Table S2**).

The prognostic values of both methods were also investigated in the multivariate Cox regression analysis to include routine clinicopathological parameters including age, treatment type, pathological lymph node status, pathological tumor size, and histological grade in the analysis. We found that while LumB_i_ patients had 2.14-fold higher risk of death than LumA_i_ from the surrogate assay (HR: 2.14, 95% CI, 0.89-5.11, *p* = 0.0873), it is not statistically significant. On the other hand, LumB_q_ specimens had 6.89 fold higher risk of death than LumA_q_ by adjusted surrogate assay (HR: 6.89, 95% CI, 2.66–17.84, *p* = 0.0001) (**Supplementary Table S3**). In addition, in both analyses, age and pathological lymph node status were found to be an independent prognostic factor.

Next, we tried to understand what caused this difference by comparing the luminal A-like and Luminal B-like subtypes from surrogate assay (LumA*_i_* and LumB*_i_*) with those from adjusted surrogate assay (LumA*_q_* and LumB*_q_*) in **Supplementary Table S4**. The specimens were named A_i_A_q_ or B_i_B_q_ if they were assigned to Luminal A-like or Luminal B-like subtypes by both methods. Those assigned by the surrogate assay to A-like subtype, but not by the adjusted surrogate assay, were named A_i_B_q_, and those assigned by the adjusted surrogate assay to Luminal-A like subtype, but not by the surrogate assay, were named B_i_A_q_. We found that more specimens were assigned to the Luminal A-like subtype by the adjusted surrogate assay than the surrogate assay (76 *vs*. 66). The overall concordance rate between the surrogate assay and the adjusted surrogate assay was 75.5%.

**Figure 4:**
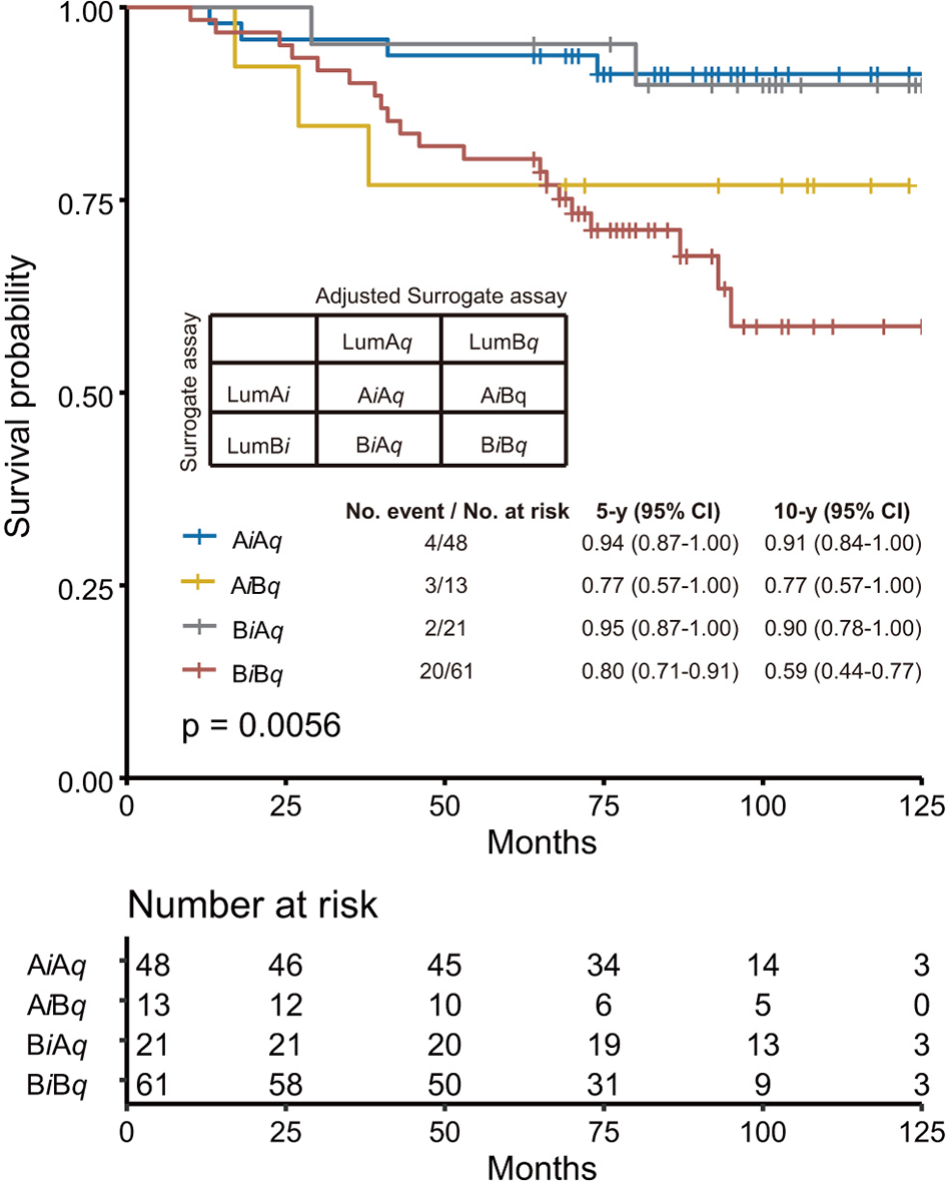
Comparison of the performance of surrogate assay with that of adjusted surrogate assay. The specimens were further subgrouped into A_i_A_q_ and B_i_B_q_ subgroups, representing specimens assigned as Luminal A-like subtype and Luminal B-like subtype by both assays; A_i_B_q_, representing specimens assigned as Luminal A subtype by surrogate assay, but as Luminal B subtype by adjusted surrogate assay; and B_i_A_q_, representing specimens assigned as Luminal B-like subtype by surrogate assay, but as Luminal A-like subtype by adjusted surrogate assay. The overall survival analysis was performed with these four subgroups using Kaplan–Meier survival analysis, with survival probability for each individual subgroup provided in the figure. The *p*-value was calculated with Log rank test.

In **Figure 4**, we performed the survival analyses of these four subgroups using Kaplan–Meier survival analysis. The A_i_A_q_ subgroup was found to have the best 10y SP at 91% *vs*. the B_i_B_q_ subgroup at 59%. In addition, the 10y SP of B_i_A_q_, the subgroup assigned to the Luminal A-like subtype only by the adjusted surrogate assay, was very close to that of A_i_A_q,_ at 90%.

### Adjusted Surrogate Assay *vs*. Surrogate Assay by Various Factors

We also attempted to minimize the influence of the type of treatment on the survival probability of each subtype (**Supplementary Figure S6**). For this purpose, only patients receiving chemotherapy were analyzed (*n* = 85), as there were an insufficient number of specimens receiving other treatments. Consistent with the overall performance, the adjusted surrogate assay presented significantly better prognosis than the surrogate assay, with 10y SP at 100% for LumA*_q_ vs*. 53% for LumB*_q_*, *p* < 0.0001, in comparison to 94% for LumA*_i_ vs*. 69% for LumB*_i_*, *p* = 0.037 (**Supplementary Figures S6A, B**).

The potential influence of pathological lymph node status was also investigated in this study by dividing patients into the pN0 group (no positive lymph node detected, *n* = 65) and pN1 (patients with 1 to 3 positive lymph nodes, *n* = 56) and analyzing their 10y SP using Kaplan–Meier analysis (**Supplementary Figure S7**). Again, the adjusted surrogate assay showed better prognosis than the surrogate assay in both cases. For pN0 patients, 10y SP was at 97% for LumA*_q_ vs*. 72% for LumB*_q_*, *p* = 0.023, in contrast to 93% for LumA*_i_ vs*. 80% for LumB*_i_*, *p* = 0.31. Likewise, this number became 90% *vs*. 63% for LumA*_q_ vs*. LumB*_q_*, *p* = 0.026, in contrast to 90% *vs*. 66% for LumA*_i_ vs*. LumB*_i_*, *p* = 0.10 for pN1 patients. The specimen numbers for pN2 and pN3 statuses were insufficient for further survival analysis.

## Discussion

In this study, by using objectively quantitated Ki67 protein levels to replace Ki67 score in the surrogate assay, we showed that inherent subjectivity and inconsistency of IHC analysis limited significantly the performance of the surrogate assay for Luminal-like breast cancer patients. A revised surrogate assay was proposed to use absolutely quantitative Ki67 levels, instead of Ki67 scores from IHC analysis, for subtyping Luminal-like patients. Upon further validation, its implementation may significantly improve the accuracy and consistency of the surrogate assay in daily clinical practice.

The standardization, or lack of standardization of Ki67 in clinical practice, is a challenge facing the whole medical community. Yet, until now, no significant progress has been made so far (10, 11, 13, 20). The significantly improved prognosis in the adjusted surrogate assay, on the other hand, suggested that QDB method may be a better option for Ki67 standardization in daily clinical practice.

One culprit underlying the lack of standardization of Ki67 scores with the IHC method is the widespread tumor heterogeneity. As the solution, the whole tissue is homogenized in the QDB method to reduce its influence to the minimum. Although the morphological features were lost in the QDB process, results from our studies suggest that the overall benefits well justify this cost in daily clinical practice.

The performance of the surrogate assay is also seriously affected by the subjectivity of the assay. We tried to overcome its influence by requesting three pathologists to judge the same set of IHC-stained slides with Ki67 independently and blindly. The IHC results from these three pathologists were analyzed with Fleiss Kappa correlation analysis with *к* = 0.633. The Ki67 scores from these three pathologists were also used to assign these specimens into Luminal A-like and Luminal B-like subtypes, respectively, with 14% as cutoff. We obtained *p*-values at 0.2, 0.018, and 0.1, respectively, with Log rank test.

Perceivably, by including more pathologists in the analysis, the subjectivity of IHC analysis should be minimized. This assumption may find its support in **Figure 1D**, where we showed significantly increased correlation between QDB and IHC when the subgroup averages of Ki67 levels from the QDB method were used in our correlation analysis. Nonetheless, this requirement will inevitably place unbearable burden to the pathologists worldwide.

As the solution, the QDB-based immunoassays provided objective and quantitative measurement of Ki67 levels, safeguarded with multiple controls. The adoption of this method may translate into significance improved consistency and reliability of the results in daily clinical practice, especially in resource-limiting laboratories where IHC analysis remains a technical challenge.

Caution should be warranted for this study for multiple reasons. First, this is only a pilot retrospective study aiming to evaluate the feasibility of the QDB method in daily clinical practice. The sample size is limited. It remains questionable if this conclusion can be held up with more FFPE specimens in the study. Second, we were unable to evaluate the performances of the surrogate assay and adjusted surrogate assay on the prognosis of other clinical outcomes, including recurrence of the disease, or disease-free survival (DFS) for lack of relevant data. Third, this study is based on real-world data of patients administered to a local hospital in China between 2008 and 2013. There were a large number of patients (14%) with no treatments documented on record for various reasons, including informed refusal or using traditional Chinese medicine. The treatments patients received were also not up to date, as reflected by the overwhelming number of Luminal-like patients receiving chemotherapy only. It should be noted that these treatments were largely following guidance issued by the China Anti-Cancer Association (CACA) in 2007 (21). All these factors may discount the conclusion of the current study. Clearly, a much larger-scale study, possibly prospective, should be carried out before the current adjustment can be considered for routine clinical practice. Nonetheless, our study suggested a potential approach to improve the performance of surrogate assay in daily clinical practice worldwide.

We also recognized the need to validate our proposed 2.31 nmol/g cutoff in an independent cohort. The effectiveness of this proposed cutoff was validated in an independent cohort of Luminal-like breast cancer specimens from another hospital alone, and in combination with the current cohort (23). We also managed to split the current cohort randomly into both trial and validation groups to demonstrate that 2.31 nmol/g was the optimized cutoff for both groups (**Supplementary Figure S4**).

Our study also hinted that the discordance between surrogate assay and genetic assays may be smaller than we expect. The discrepancy between intrinsic subtyping and surrogate assay is clearly recognized in the field (24, 25). That is also the driving force for the campaign of universal genetic testing for breast cancer patients. However, in this study, by merely improving the accuracy of Ki67 measurement in the surrogate assay, we have significantly improved the performance of the surrogate assay. Future studies are urgently needed to compare various genetic assays including PAM50 with the adjusted surrogate assay.

## Conclusions

In summary, the Ki67 protein levels were measured unprecedentedly in 253 FFPE specimens absolutely, quantitatively, and objectively using the QDB method. The measured levels were used to replace the Ki67 scores from IHC analysis in the surrogate assay, using 2.31 nmol/g as cutoff, to significantly improve the prognosis of OS in Luminal-like patients. We propose QDB as a potential solution for standardization of Ki67 assessment in daily clinical practice to improve the performance of the surrogate assay for breast cancer patients.

## Data Availability Statement

The raw data supporting the conclusions of this article will be made available by the authors, without undue reservation.

## Ethics Statement

All the studies were performed in accordance with the Declaration of Helsinki, and were approved by the Medical Ethics Committee of Yantai Affiliated Hospital of Binzhou Medical University (Approval #: 20191127001), with an informed Consent Forms waiver for archived specimens.

## Author Contributions

JH and JRZ provided clinical samples. JH, JRZ, SX, and LJ performed IHC analyses. JH supervised all the clinical studies. YL and JBZ performed all the statistical analysis. YL, YZ, FT, JL, and WZ performed all the assays and performed data analysis. XW and KC performed data analysis. JDZ designed and supervised the overall study and drafted the manuscript. JH, YL, YZ, and JDZ contributed to data interpretation and edited the manuscript. All authors contributed to the article and approved the submitted version.

## Funding

This study is sponsored by Quanticision Diagnostics, Inc.

## Conflict of Interest

YL, YZ, FT, WZ, JL, JBZ, and JDZ are employees of Yantai Quanticision Diagnostics, Inc., a division of Quanticision Diagnostics, Inc., who owned the patents covering QDB plate, QDB method, and QDB application in clinical diagnostics.

The remaining authors declare that the research was conducted in the absence of any commercial or financial relationships that could be construed as a potential conflict of interest.

The authors declare that this study received funding from Quanticision Diagnostics, Inc. The funder was involved in the study design, collection, analysis, interpretation of data, the writing of this article and the decision to submit it for publication.

## Publisher’s Note

All claims expressed in this article are solely those of the authors and do not necessarily represent those of their affiliated organizations, or those of the publisher, the editors and the reviewers. Any product that may be evaluated in this article, or claim that may be made by its manufacturer, is not guaranteed or endorsed by the publisher.

## Abbreviations

QDB, Quantitative dot blot method; FFPE, formalin-fixed paraffin-embedded; IVD, *in vitro* diagnostics; ER, estrogen receptor; PR, progesterone receptor; Her2, human epidermal growth factor receptor 2; IHC, immunohistochemistry; OS, overall survival.
