# Supplementary material for: Improving Prognosis of Surrogate Assay for Breast Cancer Patients by Absolute Quantitation of Ki67 Protein Levels Using Quantitative Dot Blot (QDB) Method
Source: Front Oncol. 2021 Sep 17;11:737781. doi: 10.3389/fonc.2021.737781 (PMC8485584; doi:10.3389/fonc.2021.737781)
Supplement: Supplementary file 1 [file DataSheet_1.doc]

**1 Supplementary Figures**


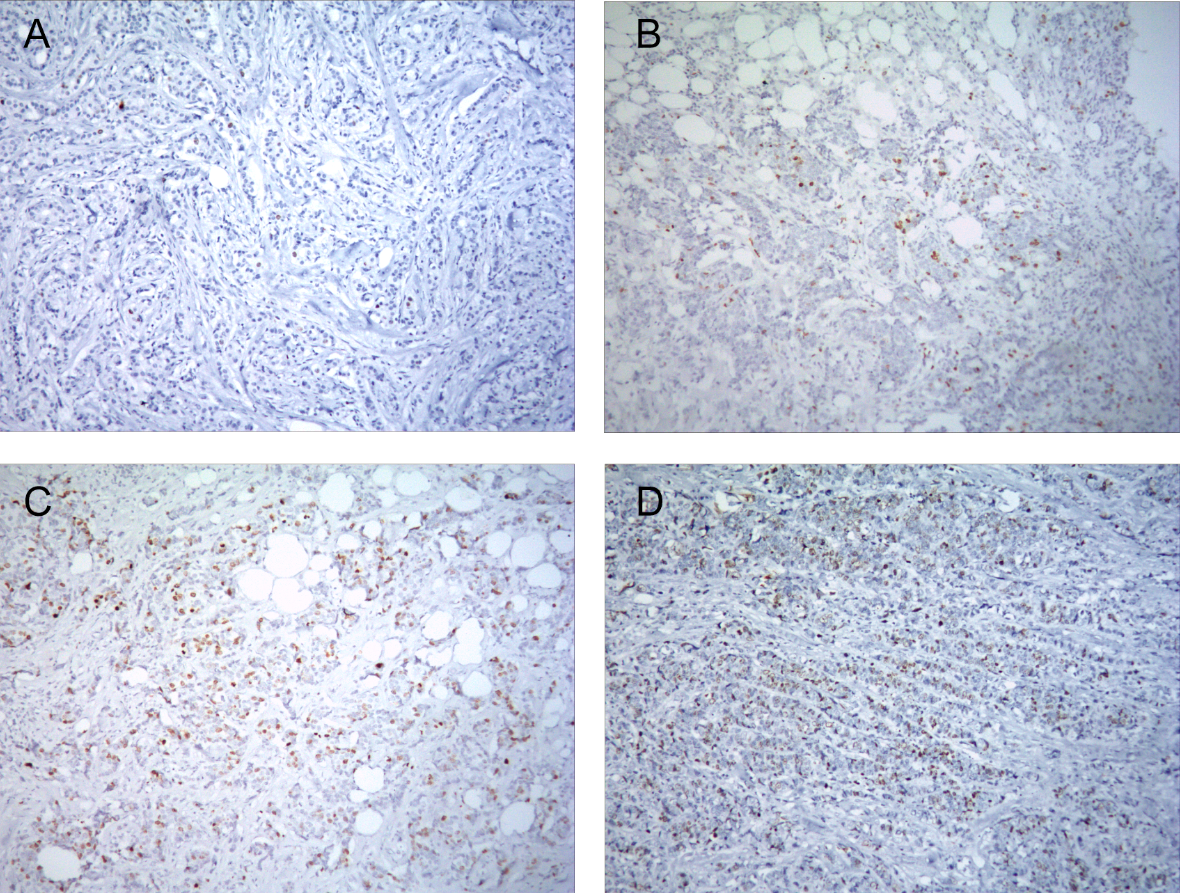


**Figure S1 A panel of typical Ki67 immunostaining images of Luminal-like breast cancer tissues sections, with (A) at 2%, (B) at 15%, (C) at 30%, & (D) at 60%**


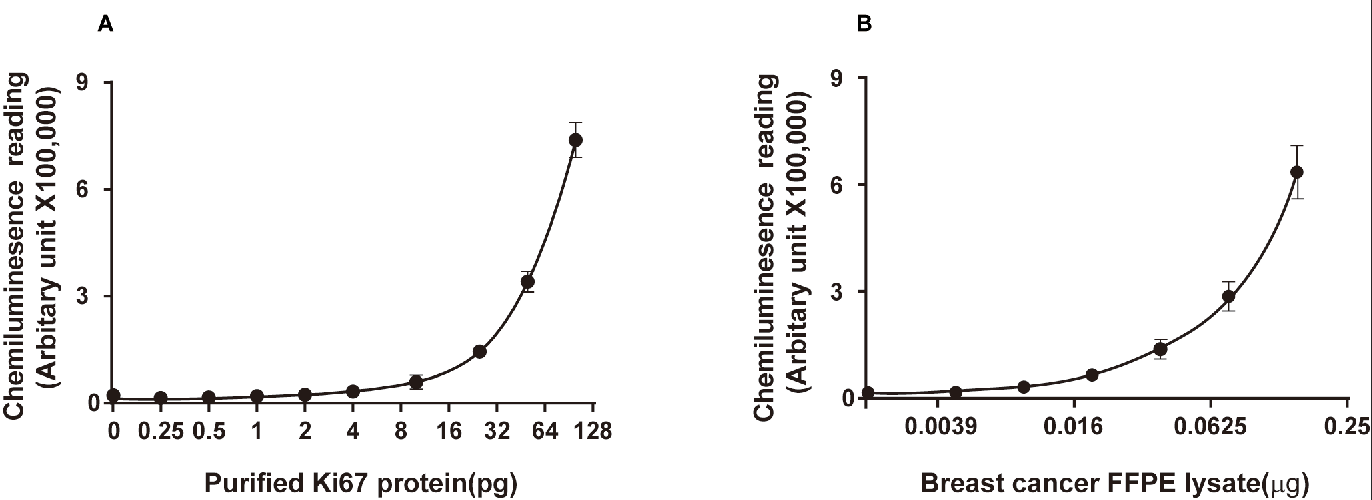


**Figure S2 Signal-to-noise ratio curves for QDB-based Ki67 immunoassay. (A), recombinant Ki67 protein; (B), breast cancer FFPE lysate**

Recombinant Ki67 protein and breast cancer FFPE lysate were serially diluted and loaded at 2 μl/unit for QDB analysis in octuplicate at each concentration except blank, which was in multiple replicates (n=20). Results were expressed as fold of signals at each concentration in log scale over that of blank.


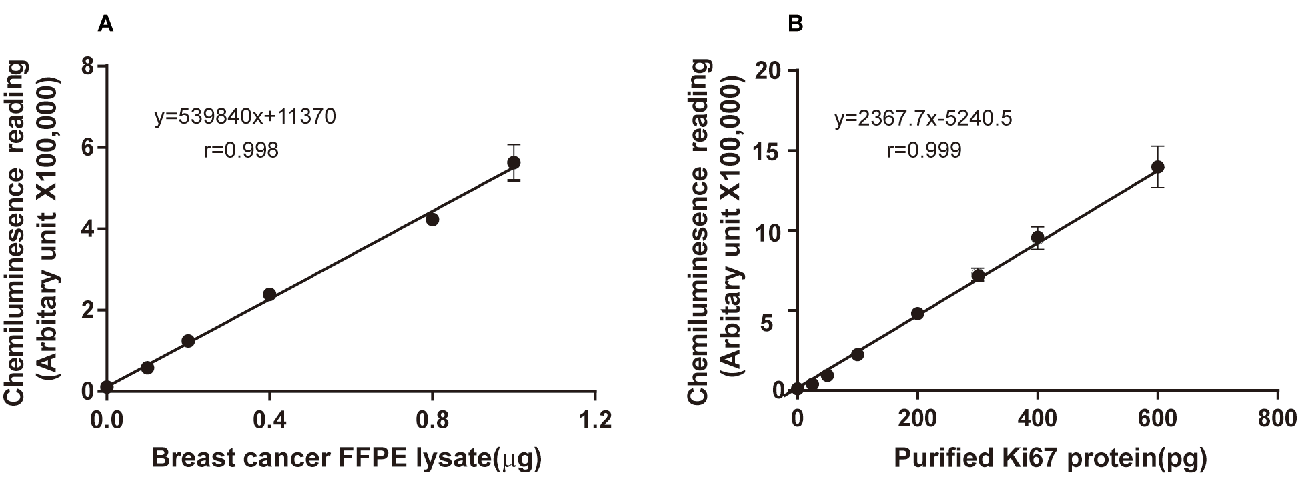


**Figure S****3 Defining the linear range of QDB measurement with Ki67 (clone MIB1)**

**(A)**, Pooled specimen prepared from 2X15 μm FFPE slices obtained from 4 patients testing positive based on IHC analyses was used to define the linear range of QDB method for Ki67.  **(B)**, Recombinant protein purified in the house was loaded at 2 μl/unit to obtain the linear range of QDB measurement. The linear range of the analysis was defined as the region where the coefficient of determination (r) was above 0.99.


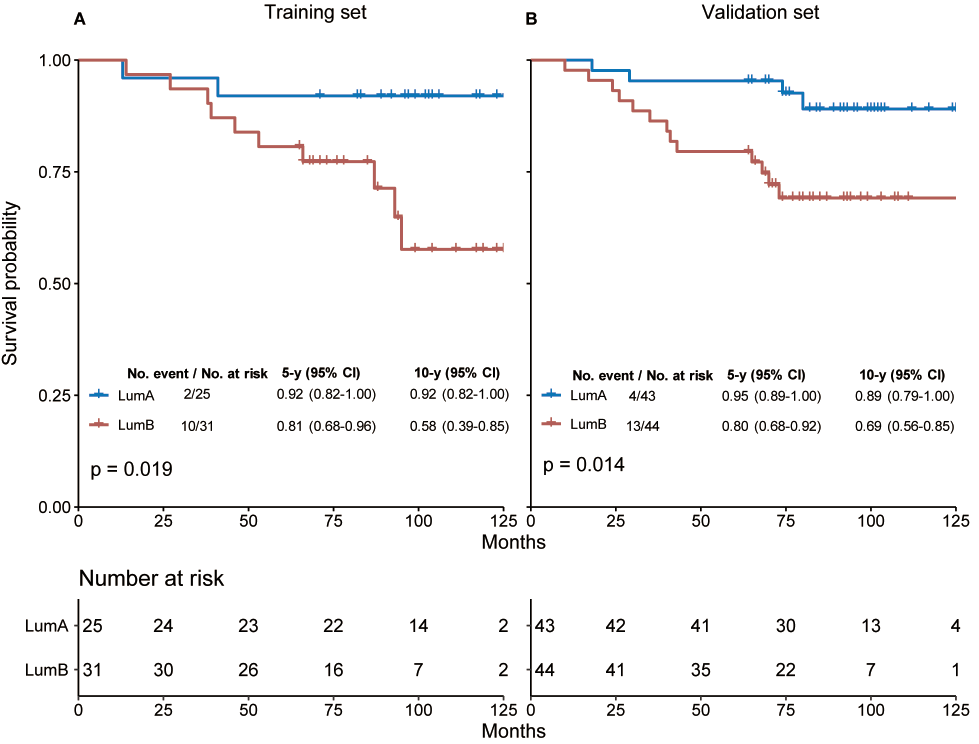


**Figure S4 Kaplan-Meier curves for OS of training and validation cohorts randomly split from specimens used in current study using Rand function of SAS 9.4**

The Luminal-like breast cancer specimens were randomly split into training and validation set using call routines and rand function in SAS 9.4 at 45% and 55% proportion. Cutoff at 2.28 nmol/g was identified using the “surv_cutpoint” function of the training set to stratify specimens for Kaplan-Meier OS analysis.


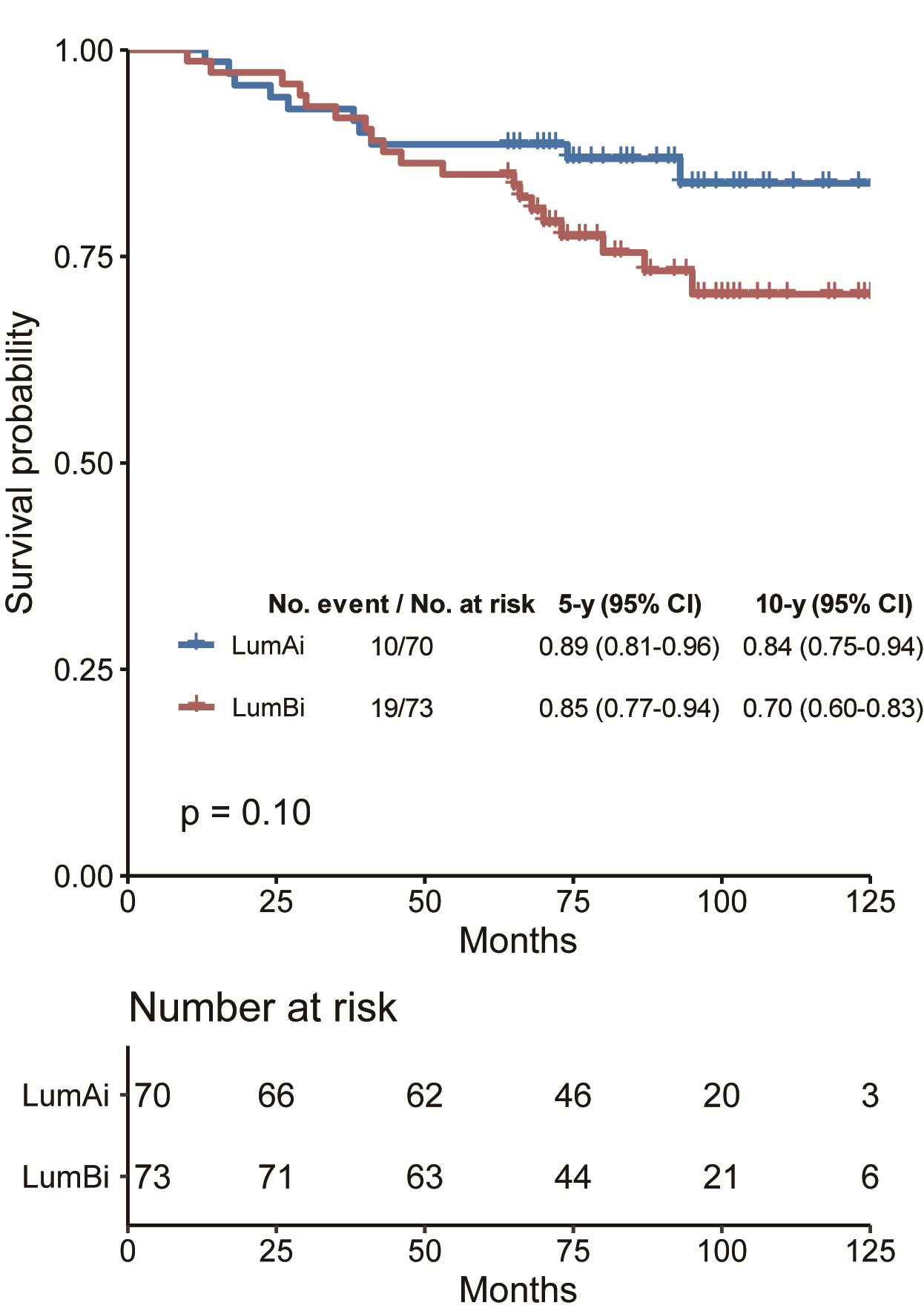


**Figure S5 Kaplan-Meier curves for OSaccording to subtypes based on 2015 St Gallen Consensus.**

Based on the 2015 St Gallen Consensus (Coates, et al, Ann Oncol. 2015: 26:1533), the cutoff for IHC-based Ki67 score was set at 20%. Patients were stratified accordingly based on their Ki67 scores, and their 5-year and 10-year survival probabilities were analyzed using Kaplan-Meier analysis. The p value was calculated using Log Rank test. LumA*_i_* and LumB*_i_*, Luminal A-like and B-like subtypes by IHC-based surrogate assay; CI, conﬁdence interval.


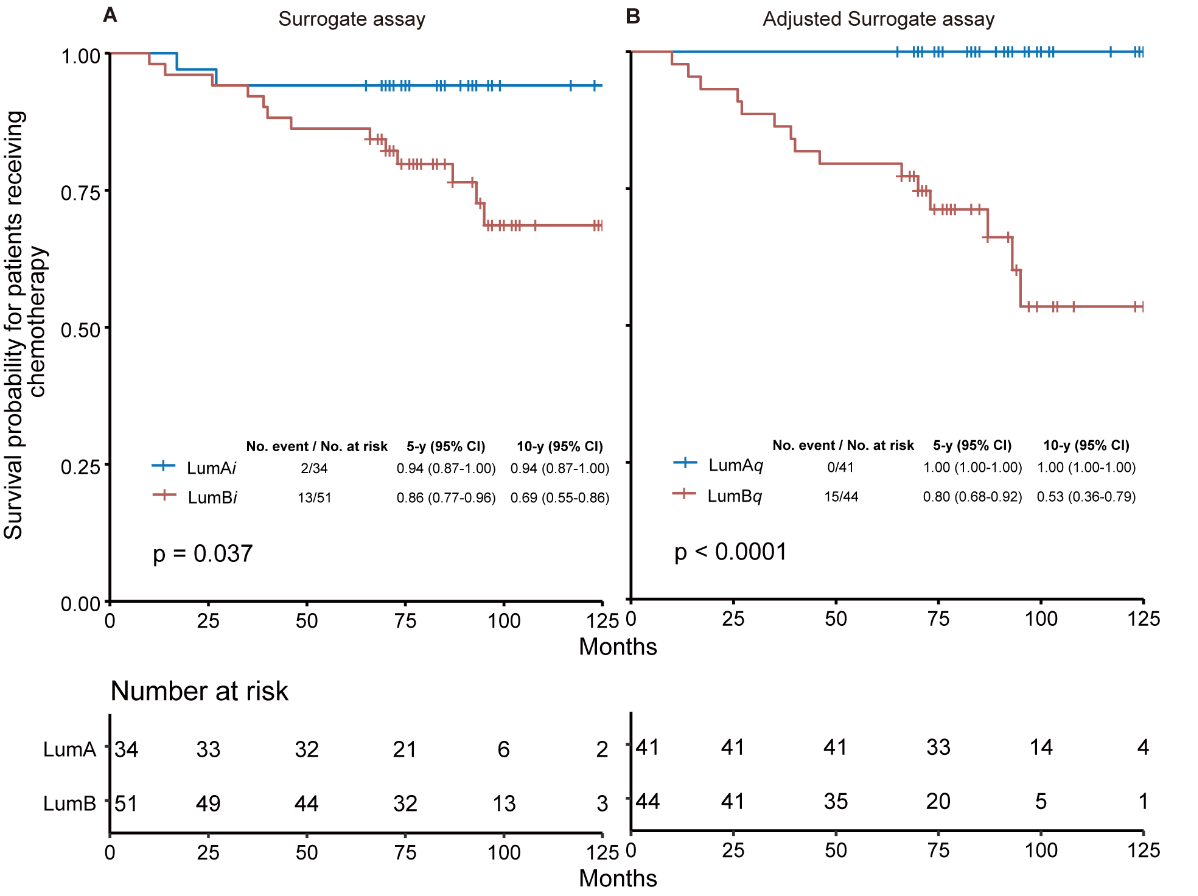


**Figure S6 *Comparison of performances of surrogate assay and adjusted surrogate assay for patients receiving chemotherapy (CT) alone***

The FFPE specimens from patients receiving chemotherapy (CT) were further subtyped into Luminal A-like and Luminal B-like subtypes using surrogate assay and adjusted surrogate assay respectively. The Kaplan-Meier survival analyses were performed for these four subtyping groups respectively, with 5-year and 10-year survival probabilities provided in the figure. The p values were calculated using Log Rank test. LumA, Luminal A-like subtype; LumB, Luminal B-like subtype; LumA*_i_* and LumB*_i_*, Luminal A-like and B-like subtypes by surrogate assay; LumA*_q_* and LumB*_q_*, Luminal A-like and B-like subtypes by adjusted surrogate assay; CI, conﬁdence interval; p, log-rank p-value.


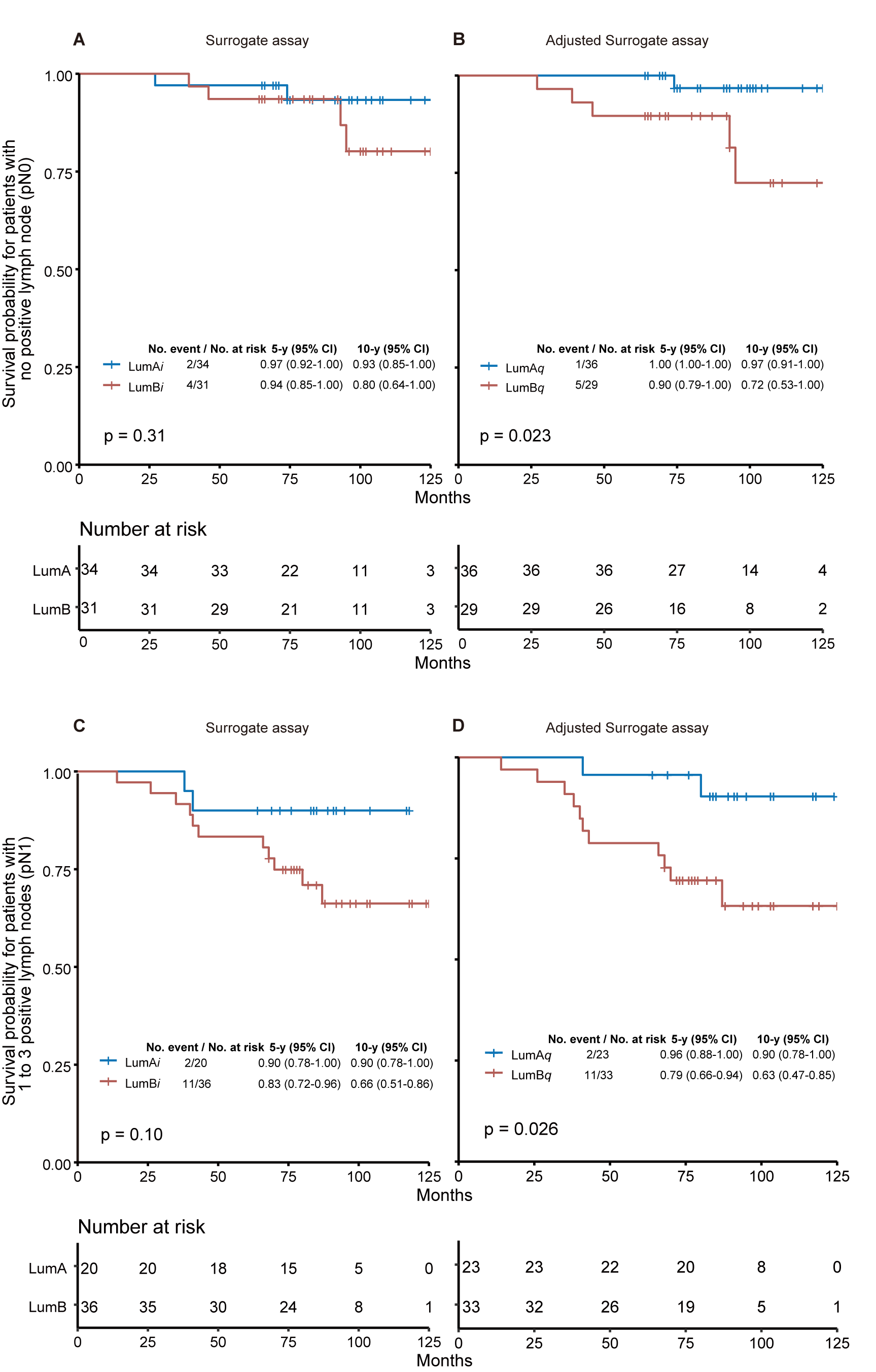


**Figure S7 *Comparison of the performances of surrogate assay and adjusted surrogate assay by pathological lymph node status, pN***

The FFPE specimens were grouped into pN0 (patients with no positive lymph node) and pN1 (patients with 1 to 3 positive lymph nodes) groups respectively. Within each group, these specimens were further subtyped into Luminal A-like and Luminal B-like subtypes using surrogate assay **(A & C)** and adjusted surrogate assay **(B & D)** respectively. The Kaplan-Meier survival analyses were performed for these four subtyping groups respectively, with 5-year and 10-year survival probabilities provided in the figure. The p values were calculated using Log Rank test. LumA, Luminal A-like subtype; LumB, Luminal B-like subtype; LumA*_i_* and LumB*_i_*, Luminal A-like and B-like subtypes by surrogate assay; LumA*_q_* and LumB*_q_*, Luminal A-like and B-like subtypes by adjusted surrogate assay; CI, conﬁdence interval; p, log-rank p-value.

**2 Supplementary Tables**

**Supplementary Table 1 Subtyping of Luminal-like specimens**

| **Subtype** | **Surrogate Assay** | **Adjusted Surrogate Assay** |
| --- | --- | --- |
| Luminal A (LumA) | ER+, Her2-, **Ki67<14%** & PR≥20% | ER+, Her2-, **Ki67<2.31nmol/g** & PR≥20% |
| Luminal B (LumB) |  |  |
| LuminalB_1_ (LumB_1_) | ER+, Her2-, **Ki67≥14%** or PR<20% | ER+, Her2-, **Ki67≥2.31nmol/g** or PR<20% |
| LuminalB_2_ (LumB_2_) | ER+, Her2+ | ER+, Her2+ |

**Supplementary Table 2 Univariate cox regression analysis of OS by surrogate assay and adjusted surrogate assay respectively**

| **Variable** | **HR** | **95%CI** | **P-value** |
| --- | --- | --- | --- |
| Surrogate Assay | 2.46 | 1.05-5.75 | 0.0385 |
| Adjusted Surrogate Assay | 4.39 | 1.78-10.81 | 0.0013 |

The 155 Luminal-like patients were subtyped based on Ki67 scores from IHC analysis (surrogate assay) or absolute Ki67 levels from QDB analysis (adjusted surrogate assay) respectively, and univariate cox regression analysis for OS was performed for these two subtyping methods respectively.

**Supplementary Table 3 Multivariate cox regression analysis of OS by surrogate assay and adjusted surrogate assay respectively**

| **Variable** | **Surrogate Assay** | | |  | **Adjusted Surrogate Assay** | | |
| --- | --- | --- | --- | --- | --- | --- | --- |
|  | **HR** | **95%CI** | **P-value** |  | **HR** | **95%CI** | **P-value** |
| Age | 3.53 | 1.41-8.80 | 0.0069 |  | 5.79 | 2.22-15.10 | 0.0003 |
| Treatment Type^a^ | 1.15 | 0.70-1.91 | 0.5759 |  | 1.06 | 0.64-1.76 | 0.8257 |
| Pathological Lymph Node Status, pN | 1.67 | 1.25-2.23 | 0.0006 |  | 1.86 | 1.36-2.54 | 0.0001 |
| Pathological Tumor Size, pT | 1.19 | 0.60-2.37 | 0.6191 |  | 1.33 | 0.61-2.88 | 0.4729 |
| Histological Grade | 0.97 | 0.53-1.76 | 0.9144 |  | 0.88 | 0.47-1.66 | 0.7006 |
| Surrogate Assay | 2.14 | 0.89-5.11 | 0.0873 |  | - | - | - |
| Adjusted Surrogate Assay | - | - | - |  | 6.89 | 2.66-17.84 | 0.0001 |

^a^ a disordered categorical variable of endocrinotherapy (ET), chemotherapy (CT), endocrinotherapy & chemotherapy (CET) and others to be assigned as 0, 1, 2, 3 respectively for statistical analysis.

The 155 Luminal-like patients were subtyped based on surrogate or adjusted surrogate assay respectively, and analyzed together with age, treatment, pathological lymph node status, pathological tumor size, and histological grade in multivariate cox regression analysis for OS of these patients.

**Supplementary Table 4 Concordance of surrogate assay and adjusted surrogate assay**

| **Surrogate Assay** | **Adjusted Surrogate Assay** | | **Total** |
| --- | --- | --- | --- |
|  | **LumA*_q_*** | **LumB*_q_*** |  |
| **LumA*_i_*** | 52 (A*_i_*A*_q_*) | 14 (A*_i_*B*_q_*) | 66 |
| **LumB*_i_*** | 24 (B*_i_*A*_q_*) | 65 (B*_i_*B*_q_*) | 89 |
| **Total** | 76 | 79 | 155 |

The 155 Luminal-like patients were subtyped into 66 Luminal A-like (LumA*_i_*) and 89 Luminal B-like (LumB*_i_*) subtypes based on surrogate assay, or 76 Luminal A-like (LumA*_q_*) and 79 Luminal B-like (LumB*_q_*) subtypes based on adjusted surrogate assay. There were 52 patients assigned by both surrogate assay and adjusted surrogate assay as Luminal A-like subtype (A_i_A_q_), 65 assigned by both surrogate assay and adjusted surrogate assay as Luminal B-like subtype (B_i_B_q_), 14 assigned by surrogate assay as Luminal A-like subtype, but assigned as Luminal B-like subtype by adjusted surrogate assay (A_i_B_q_), and 24 assigned by surrogate assay as Luminal B-like subtype, but as Luminal A-like subtype by adjusted surrogate assay (B_i_A_q_). The overall concordance of these two methods were (52+65)/155=75.5%.
